# Profiling of volatile fragrant components in a mini-core collection of mango germplasms from seven countries

**DOI:** 10.1371/journal.pone.0187487

**Published:** 2017-12-06

**Authors:** Li Li, Xiao-Wei Ma, Ru-Lin Zhan, Hong-Xia Wu, Quan-Sheng Yao, Wen-Tian Xu, Chun Luo, Yi-Gang Zhou, Qing-Zhi Liang, Song-Biao Wang

**Affiliations:** Key Laboratory of Tropical Fruit Biology of Ministry of Agriculture, South Subtropical Crops Research Institute, Chinese Academy of Tropical Agricultural Sciences, Zhanjiang, China; United States Department of Agriculture, UNITED STATES

## Abstract

Aroma is important in assessing the quality of fresh fruit and their processed products, and could provide good indicators for the development of local cultivars in the mango industry. In this study, the volatile diversity of 25 mango cultivars from China, America, Thailand, India, Cuba, Indonesia, and the Philippines was investigated. The volatile compositions, their relative contents, and the intervarietal differences were detected with headspace solid phase microextraction tandem gas chromatography-mass spectrometer methods. The similarities were also evaluated with a cluster analysis and correlation analysis of the volatiles. The differences in mango volatiles in different districts are also discussed. Our results show significant differences in the volatile compositions and their relative contents among the individual cultivars and regions. In total, 127 volatiles were found in all the cultivars, belonging to various chemical classes. The highest and lowest qualitative abundances of volatiles were detected in ‘Zihua’ and ‘Mallika’ cultivars, respectively. Based on the cumulative occurrence of members of the classes of volatiles, the cultivars were grouped into monoterpenes (16 cultivars), proportion and balanced (eight cultivars), and nonterpene groups (one cultivars). Terpene hydrocarbons were the major volatiles in these cultivars, with terpinolene, 3-carene, caryophyllene and α-Pinene the dominant components depending on the cultivars. Monoterpenes, some of the primary volatile components, were the most abundant aroma compounds, whereas aldehydes were the least abundant in the mango pulp. β-Myrcene, a major terpene, accounted for 58.93% of the total flavor volatile compounds in ‘Xiaofei’ (Philippens). γ-Octanoic lactone was the only ester in the total flavor volatile compounds, with its highest concentration in ‘Guiya’ (China). Hexamethyl cyclotrisiloxane was the most abundant volatile compound in ‘Magovar’ (India), accounting for 46.66% of the total flavor volatiles. A typical aldehydic aroma 2,6-di-tert-butyl-4-sec-butylphenol, was detected in ‘Gleck’. A highly significant positive correlation was detected between Alc and K, Alk and Nt, O and L. Cultivars originating from America, Thailand, Cuba, India, Indonesia and the Philippines were more similar to each other than to those from China. This study provides a high-value dataset for use in development of health care products, diversified mango breeding, and local extension of mango cultivars.

## Introduction

Aroma is an important attribute, reflecting fruit quality, ripeness, and consumer acceptance [1–4]. Aroma is conferred by a range of diverse low-molecular-weight, volatile,chemical compounds that occur widely in nature. It plays a key role in the quality profiles of fruit (including mango, apple, grape, etc.) and is an attribute considered in flavor and fragrance production not only in the food industry but also in the cosmetics industry. Monoterpenes, sesquiterpenes, esters, lactones, alcohols, aldehydes, ketones, volatile fatty acids, some degradation product of phenols, and some carotenoid contributes to the aromatic volatile profile of mango fruit [5–10]. Aroma is also important in assessing the quality of fresh mango and its processed products, and is a good index for screening mango germplasm resources for the development of local cultivars.

Mango (*Mangifera indica* L.), often called the “king of fruits”, is the most popular fruit throughout the world in terms of its production, marketing and consumption [11,12]. Among tropical fruits, it ranks second only to the banana in international trade [13]. Mango production throughout the world is nearly 42.66 million tones, and China is the second largest mango-producing country, producing slightly less than 4.62 million tonnes in 2013 [14]. Apart from the consumption of fresh mangoes, mango pulp and concentrated juice are widely traded in current international markets as a base material in the beverage industry and as a flavoring ingredient in the dairy industry and baby food formulations [14].

Aroma is specific to each mango cultivar [7]. ‘Alphonso’ is a unique mango cultivar with an abundance of both lactones and furanone in its ripe fruits [9,11,15]. δ-3-carene as the only compound that contributes the mango-like notes to western mango cultivars [8]. This observation is supported by a subsequent finding that non-Indian cultivars invariably contain δ-3-carene as their principle volatile component [9,11]. Sixty-one aromatic volatile compounds were identified in the ‘Kensington Pride’ mango by Lalel et al. [7], of which 35 had not been reported previously. Lalel et al. [16] also measured Glycosidically bound aromatic volatile compounds in the skin and pulp of the ‘Kensington Pride’ mango fruit at different stages of maturity, and found that only terpenes seem to contribute to the aroma of fresh ripe ‘Kensington Pride’ mangoes.

The composition and concentrations of aromatic volatiles in mango fruit are influenced by the cultivar [9,10]. Pandit et al.[9] analyzed 27 mango cultivars, detecting 84 volatile compounds, and the highest and lowest qualitative abundances of volatiles were detected in the ‘Alphonso’ and ‘Pairi’ cultivars, respectively. John et al. [17] found differences in the compositions and concentrations of terpenoid compounds in the sap of seven Indian mango varieties, including monoterpenes β-myrcene, trans-/cis-ocimene, and limonene. Based on a study that revealed significant differences in the fresh flavors and textures of six Thai mangoes, Ledeker et al. [18] suggested that manufacturers should select the appropriate cultivars for mango purées because heat treatment significantly changes the flavor and texture, Musharraf et al. [12] developed a quantitative method based on gas chromatography-triple quadrupole mass spectrometry for the analysis of the aroma component of mango sap in nine Pakistani cultivars, and identified seven terpenes, α-pinene, α-phellandrene, (+)-3-carene, sabinene, γ-terpinene, (-)-trans-caryophyllene, and α-humulene. When analyzing the aromas of 15 varieties of mango cultivated in Brazil, Andrade et al. [19] found that α-terpinolene was the principal constituent of the ‘Willard’, ‘Parrot’, ‘Bowen’ and ‘Kensington’ varieties; α-terpinolene and Δ3-carene mainly occurred in Sri Lanka varieties; Δ3-carene was the major contributor to the aroma of mango fruit grown in Venezuela and Myrcene was characteristic of the ‘Alphonso’ variety from India.

Although mango germplasm resource are relatively abundant throughout the world, even in China, mango breeding objectives largely focus on disease resistance, or fruit size, sweetness, or color [20], rather than on aroma quality. Currently, artificial breeding that has ignored the properties of fruit aroma, making mango cultivars lack aroma diversification and resource superiority has been not take full their advantage. The main constraint is that the aroma properties of mango germplasms are unclear. Therefore, we aimed at improving the database of mango germplasm resource to provide a basis for breeding unique-functional mango cultivars, to promote the classification of varieties. Twenty-five mango cultivars, either cultivated in China or indigenous cultivars from more than seven states or districts, were qualitatively and quantitatively analyzed with solid-phase microextraction-gas chromatography-tandem mass spectrometer (SPME-GC-MS). The volatile component profiles of all the mango cultivars are described and the cultivars were classified according to a cluster analysis based on their volatile species.

## Materials and methods

### Plant material, sampling, and chemicals

Twenty-five mango cultivars and their districts of origin are shown in Table 1, All were grafted on the 17-year-old rootstock Yuexi No.1. which grows in an orchard located in the Mango germplasm Nursery of the South Subtropical Crops Research Institute, Chinese Academy of Tropical Agricultural Sciences in Zhanjiang, Guangdong Province, China. For each cultivar, night trees of similar size and similar vigor were randomly selected for sampling. Of these, each three trees consisting of a replicate from which 15–20 mango fruits were sampled from the top, center, and bottom of the trees.

**Table 1:** Mango cultivars analyzed in this study and their origins.

Origin	Cultivar
China	Zihua(1), Shixuan No.8(2), Renong No.1(3), Guire No.82(4), Tainong No.1(5), Hongmang No.8(6)
America	Haden (7), Tommy Atkins(8), Edward(9), Glenn(10)
Thailand	Hongmang No.11(11), Keawsaweuy(12), Choke Anand(13), Nam Dok Mai(14), Thai 506(15)
Cuba	Cuba No.1(16), Cuba No.3(17), Cubasankeli(18)
India	Mallika(19), Magovar(20), Saigon(21)
Indonesia & Philippines	Gleck(22), Baodaohuang(23), Guiya(24), Xiaofei(25)

Note: Numbers in parentheses of following the cultivar names are the accession numbers.

All chemicals and reagents for aroma extraction and the GC/MS analysis were purchased from Thermo Fisher Scientific (USA), Alfa Aesar (USA) or Sigma-Aldrich Co. (St. Louis, MO, USA) and were of the highest available purity. The SPME instrument was from Supelco Company of Sigma-Aldrich (St. Louis, MO, USA). Water was purified with a Milli-Q deionization unit (Millipore, Bedford, MA, USA). The Trace GC Ultra-ISQ MS system was from Thermo Fisher Scientific, with a gas chromatograph (Agilent 6890N) coupled to a mass spectrometer (Agilent 5975). The GC column was a fused silica capillary column (Agilent DB-5 MS, 30 m × 0.25 mm ID × 0.25 μm film thickness).

### Sample pretreatment and extraction

At the harvesting stage of maturity, fruit at about 80% full ripeness and of identical size were picked and transported immediately to the laboratory to accelerate their ripening. After about 3 days, the fruit were fully ripe and suitable for pulp collection. After the fruit were peeled and cut into small pieces, the pulp was frozen with liquid nitrogen and ground into powder. All the homogenized pulp powder were immediately stored at -40°C for subsequent analyses.

The powdered fresh pulp (8 g) was added to 25-mL vial, which was sealed with a polytetrafluoroethylene isolation membrane (Tianjin Jinteng Experiment Equipment Co., Ltd., China) to extract the aroma compounds with headspace SPME (HS-SPME). The SPME syringe (100 μm polydimethylsiloxane) was then inserted into the headspace of the vial and the volatiles were extracted at 25°C in a thermostatically controlled water bath for 40 min with continuous stirring.

### GC/MS analysis and MS library matching

GC-MS analysis was performed as described by Musharraf et al. [12] and Wei et al. [21] with modifications. After extraction, the SPME fiber was placed into the GC-MS injector and maintained for 3 min for desorption. The GC-MS system consisted of a Trace^™^ GC Ultra gas chromatograph and a tandem ISQ mass spectrometer. Chromatographic conditions were: pressure of the carrier gas (helium) of 7.7 Psi at the initial oven temperature, with flow rate 1 mL·min^-1^. All samples were injected in the splitless mode. The injector temperature was 250°C; the initial oven temperature was 50°C for 1 min, which was increased to 140°C at a rate of 5°C min^-1^, and then to 250°C at a rate of 10°C min^-1^, where it was maintained for 10 min (total run time, 45 min). The mass spectrometer was operated in electron impact (EI) mode using parameters: EI mode at 70 eV, with a scan range of 35―335 m/z. The temperatures of the transfer line and of the ion source was set to 250 and 200°C, respectively. The sample (volume 1.0 μL) was injected in triplicate throughout the study. The Mass Hunter software (Agilent) was used for data acquisition and processing. The peaks of the aromas compounds were identified by their retention times, with references to standard compounds, and the mass spectra obtained were compared with those available in the Wiley and NIST08 libraries (Wiley RegistryTM, 8th Edition Mass Spectral Library, and the NIST 08 Mass Spectral Library [NIST/EPA/NIH] 2008 version), with an acceptance criterion of a score match > 70%. Signal were acquired and the analytes were quantified with Multiple Reaction Monitoring (MRM) to determine the precursor-product ion pairs of the transitions. The relative percentage contents of all the analytes were calculated by normalizing the peak areas.

### Statistic analysis

The signal analysis and the automatic integration and generation of signal charts using the Analyst software were appropriate for the GC-MS equipment. For each component within a mango variety, the content was determined from three biology replicates and each replicate was analyzed with three technical repetitions. Microsoft Office Excel 2010 was used to calculate the target components, relative standard deviations, and bias.

In this study, all the components detected were first grouped into different compounds, according to the similarity of their chemical property. The relative contents of the compounds were subjected to a correlation analysis and a cluster analysis. The content of each compound in a replicate mango variety was generated with the formula: value-1 = Ʃ(mean scores of the three replicates of each component, including all components of the same compound from the same varieties). The value-1 data were used for a cluster analysis of the varieties, using the XLSTAT 2010 software, and to calculate Pearson’s correlation coefficients (correlation analysis) across compounds, with the SPASS (version 17.0) software. Similarly, value-2 = log _2_ (Ʃ[value-1 of each variety from the same region]/number of varieties in the corresponding region) was used for the cluster analysis across regions with HemI 1.0.3.3 software.

## Results and discussion

### Scanning of volatile composition and proportion from 25 mango pulp

**Fig 1:**
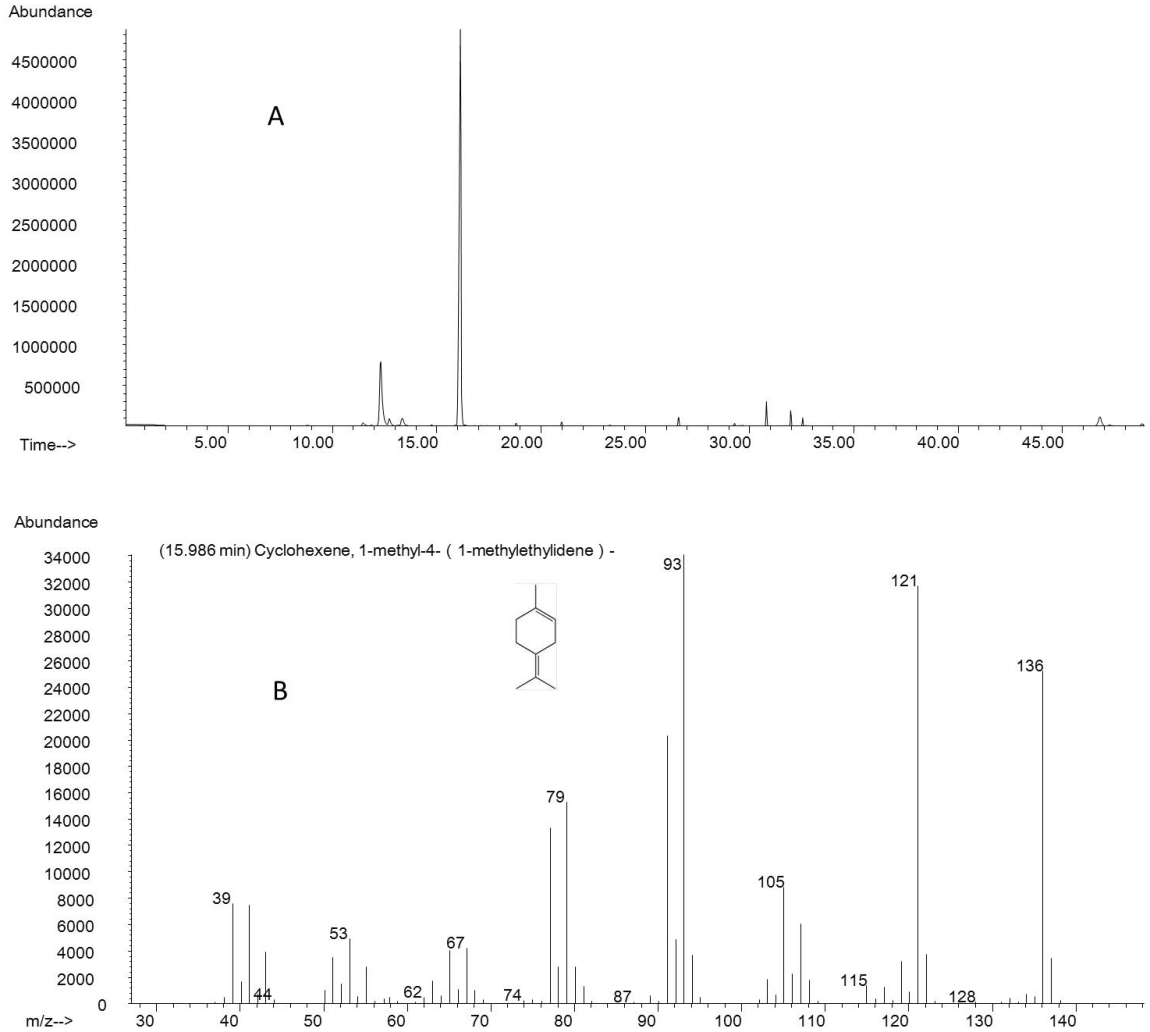
Representative GC–MS chromatogram of volatiles from mango pulp. A: Representative GC**–**MS chromatogram of a selected sample (‘Renong 1’); B: representative total ion mass spectrum of α-terpinolene (retention time, 15.986 min) from ‘Renong 1’.

The aroma components of 25 mango pulps was determined independently with HS-SPME-GC-MS. Fig 1 (A & B) show a representative GC-MS chromatogram of a selected sample (‘Renong 1’) and the total ion mass spectrum of α-terpinolene, respectively. Altogether, 127 volatile components were detected in the mangoes in this study (Table 2). The compositions and proportions of volatiles in the mango fruit of individual cultivars are also shown in Table 2. These results suggest that mango pulp is very rich in volatile components. The Thai cultivar ‘Thai 506’ had the most volatile components (32 aroma components) ranking first among all the cultivars analyzed. In contrast, the Chinese cultivar ‘Shixuan No. 8’ and the Thai cultivars ‘Hongmang No. 11’ had the least volatile components, containing only two volatile components each, terpinolene (2.41%) and squalene (14.84%) in ‘Shixuan No. 8’ and 1-Methyl-4-(1-methylethylidene)-cyclohexene (1.12%) and 3-Carene (26.35%) in ‘Hongmang No. 11’.

**Table 2:** Compositions and relative contents of all volatiles from 25 mango cultivars (%). Table in the annex.

Code	Volatile component	Cultivar																								
		1	2	3	4	5	6	7	8	9	10	11	12	13	14	15	16	17	18	19	20	21	22	23	24	25
1	α-Panasinsen															0.0312								0.0798		
2	4-α-Isopropenyl-2-carene									1.6782																
3	4-Carene					0.5576										1.6387	0.5476						81.2519			
4	Epi-bicyclosesquiphellandrene						0.0281																			
5	α-Caryophyllene	0.7902		1.3301	0.8934	0.0906		0.8239	2.5755						2.1961	0.0797	7.1135	0.2779	0.1057		1.6404		0.4192	0.0981		
6	α-Cubebene			0.2453		1.5866	0.2769		0.0347								0.026						0.0465			0.5382
7	α-Pinene								6.5207																	
8	β-Myrcene								1.0777										0.5228							58.9252
9	β-Panasinsene																									0.2599
10	β-Phellandrene																						0.6011	0.6324		
11	β-Pinene	0.7436		0.7663					0.802							2.4686							0.0433			0.6671
12	γ-Elemene												1.2064													
13	1,2-Benzenedicarboxylic acid, bis(2-methylpropyl) ester	0.0535																								
14	Diisooctyl phthalate						0.7018																			
15	1,2-Nonadiene	0.717																								
16	1,3,5,7,9-Pentaethylcyclopentasiloxane									15.3875															14.3308	
17	1,3,5,8-Undecatetraene	0.1931												0.2697												0.1105
18	2,5,5-Trimethyl-1,3,6-heptatriene												0.3421													
19	(E)-3,7-Dimethyl-1,3,6-octatriene												55.3169			5.5873					34.9883					15.4551
20	(Z)-3,7-Dimethyl-1,3,6-octatriene				71.67		0.038										0.075				9.8259		0.1453			
21	1,7-Dimethyl-1,3,7-cyclodecatriene										4.9807															
22	3,7-Dimethyl-1,3,7-octatriene										1.7927															
23	1,3,8-p-Menthatriene					0.1306										4.3817							0.0378	0.346		
24	1,3,5,5-Tetramethyl-1,3-cyclohexadiene																				0.7255					
25	1,5,5,6-Tetramethyl-1,3-cyclohexadiene															2.0304										
26	1-Methyl-4-(1-methylethyl)-1,3-cyclohexadiene			1.6878		2.2205	1.0879	2.9598	0.4117										1.4136				2.1669	2.0536		
27	3,6-Dimethyl-1-cyclohexene-1,2-dicarboxylic anhydride																	0.2495								
28	1,5,9,9-tetramethyl-1,4,7,-Cycloundecatriene						0.3483						1.209													
29	1-methyl-4-(1-methylethyl)-,1,4-Cyclohexadiene	0.4054		0.1697		0.22	0.1143		0.2657							3.9026	0.1032						0.2589			0.1764
30	1,5,5-Trimethyl-6-methylene-cyclohexene	0.0432																								
31	1-methyl-5-methylene-8-(1-methylethyl)-1,6-Cyclodecadiene	0.8607				0.1277							3.5056		7.7999		0.098					1.0609	0.0035			
32	3,4-dihydro-8-hydroxy-3-methyl-, (R)-,1H-2-Benzopyran-1-one															0.0483										
33	1H-Cyclopenta[1,3]cyclopropa[1,2]benzene, octahydro-7-methyl-3-methylene-4-(1-methylethyl)-, [3aS-(3a.alpha.,3b.beta.,4.beta.,7.alpha.,7aS*)]-1H-Cyclopenta[1,3]cyclopropa[1,2]benzene	0.0729											0.3677				0.0433									
34	1a,2,3,4,4a,5,6,7b-octahydro-1,1,4,7-tetramethyl-, [1aR-(1a.alpha.,4.alpha.,4a.beta.,7b.alpha.)]-1H-Cycloprop[e]azulene	0.0344			0.9584		0.0404		0.3654				0.3489			0.0913	0.4952	0.299								
35	1a,2,3,4,4a,5,6,7b-octahydro-1,1,4,7-tetramethyl-, [1aR-(1a.alpha.,4.alpha.,4a.beta.,7b.alpha.)]-1H-Cycloprop[e]azulene																									0.0551
36	Ledene																0.175									
37	decahydro-1,1,7-trimethyl-4-methylene-, [1aR-(1a.alpha.,4a.beta.,7.alpha.,7a.beta.,7b.alpha.)]-1H-Cycloprop[e]azulene															0.0226	0.0247							0.2985		0.1251
38	1a,2,3,3a,4,5,6,7b-octahydro-1,1,3a,7-tetramethyl-, [1aR-(1a.alpha.,3a.alpha.,7b.alpha.)]-1H-Cyclopropa[a]naphthalene	0.2361																								
39	1a,2,3,5,6,7,7a,7b-octahydro-1,1,7,7a-tetramethyl-, [1aR-(1a.alpha.,7.alpha.,7a.alpha.,7b.alpha.)]-1H-Cyclopropa[a]naphthalene	0.2464				0.1262	0.0633						0.1703				0.0313									
40	1R-α-Pinene	2.6515		0.167	1.6285	0.2793	0.2227						1.5891			3.4447		0.4983						0.2667		5.7238
41	1S-α-Pinene																0.3832		2.7223				0.3547			
42	5-butyldihydro-,2(3H)-Furanone	1.0966			1.2702											0.0234									25.8522	0.3352
43	Thiophenone						0.0162																			
44	2,6-Dimethyl-2,4,6-Octatriene,																				36.9021		0.0357			0.1736
45	2,6,6-Trimethyl-2,4-cyclohrptadien-1-one																	3.0314								
46	2,6-Bis(1,1-dimethylethyl)-4-(1-oxopropyl)phenol						0.0844																			
47	3,7-dimethyl-, (Z)-,2,6-Octadien-1-ol													0.1154												
48	Crotonic acid cis-3-hexen-1-yl- ester			0.1108																				0.1025		
49	1,3-dihydro-5-methyl-, 2H-Benzimidazol-2-one						0.0146																			
50	tetrahydro-6-propyl-,2H-Pyran-2-one	0.1407																								
51	3-Methylnorcaran-2-one																									0.0459
52	4-Methoxy-2,5-dimethyl-3(2H)-furanone				1.162																					
53	2,2,4,4,5,5,7,7-octamethyl-3,6-Dioxa-2,4,5,7-tetrasilaoctane										9.4042															
54	3,6-Dioxa-2,4,5,7-tetrasilaoctane, 2,2,4,4,5,5,7,7-octamethyl-2,2,4,4,5,5,7,7-octamethyl-,3,6-Dioxa-2,4,5,7-tetrasilaoctane										61.7749															
55	3-Carene			15.8931			13.178		56.0521	18.5973	16.0272	26.3469	4.7682	0.606			66.5493		3.5464							
56	Leaf alcohol			0.0662														0.4243								
57	di-TMS,3-Hydroxymandelic acid,ethyl ester									6.8367																
58	4-Hydroxymandelic acid, ethyl ester														4.625										7.2629	
59	1,2,3,4,5,6,7,8-octahydro-1,4-dimethyl-7-(1-methylethenyl)-, [1S-(1.alpha.,4.alpha.,7.alpha.)]-Azulene	0.3551																								0.2305
60	1,2,3,5,6,7,8,8a-octahydro-1,4-dimethyl-7-(1-methylethenyl)-, [1S-(1.alpha.,7.alpha.,8a.beta.)]-Azulene	1.3421											0.7405													0.562
61	2,5-bis[(trimethylsilyl)oxy]-Benzaldehyde			0.3364																						
62	1,2,3,4-Tetramethyl-benzene																						0.0232	0.2214		
63	2-Methyl-m-phenylene diisocyanate						0.0291																0.0038			
64	1-ethyl-2,3-dimethyl-Benzene															2.0041										
65	1-methyl-2-(1-methylethyl)-Benzene	0.1692																								
66	M-Cymene			0.1183																						
67	1-methyl-4-(1-methylethenyl)-Benzene						0.1546									3.9097		0.221								
68	1-methyl-4-(1-methylethyl)-Benzene															1.2242										0.0734
69	2,4-diisocyanato-1-methyl-Benzene						0.0456							0.0405									0.0064			
70	2-Ethyl-1,4-dimethyl—benzene																							0.1851		
71	2,3-dihydro-2-methyl-Benzofuran,					0.1161																				
72	5-methyl-2-trimethylsilyloxy-benzoic acid, trimethylsilyl ester																								10.8052	
73	Thujone																0.0387									
74	4-methyl-1-(1-methylethyl)-Bicyclo[3.1.0]hex-2-ene						0.5373										1.924									
75	α-Pinene																			0.086						
76	2,6-dimethyl-6-(4-methyl-3-pentenyl)bicyclo[3.1.1]hept-2-ene																0.0641									
77	3,6,6-trimethyl-Bicyclo[3.1.1]hept-2-ene															2.629										
78	6,6-dimethyl-2-methylene-Bicyclo[3.1.1]heptane	0.2901																								
79	3,7,7-trimethyl-Bicyclo[4.1.0]hept-2-ene	1.9211														3.132										
80	3,7,7-trimethyl-(1S)-Bicyclo[4.1.0]hept-3-ene	4.2718			11.149	5.4888		76.2961	0.3683							4.494		69.0401					4.3691	5.0789		
81	4,11,11-Trimethyl-8-methylene-Bicyclo[7.2.0]undec-4-ene																0.0214									
82	Butanoic acid,2-methylpropyl ester															1.6604										
83	(E)-Butanoic acid, 3-hexenyl ester			0.4512																						
84	(Z)-Butanoic acid, 3-hexenyl ester					0.9817							1.9066	1.7441		0.6321					0.5695		0.124	1.213		
85	Butanoic acid, hexyl ester																						0.0198			
86	Butanoic acid, octyl ester	0.0428																								
87	Camphene								0.0898							0.8166							0.0109			0.0502
88	Caryophyllene	1.3729		1.9911	1.6518	0.2664	0.7445	1.9939	5.0832				4.491		12.2009	0.1486	12.1097	0.678	0.3852		3.0453	2.1023	0.9352	0.2351		
89	Copaene	0.0626							1.6147																	
90	1-methyl-4-(1-methylethylidene)-Cyclohexene	76.0088	2.4124	69.2617	0.7227	78.3034	34.4422		4.4233			1.1237	7.5131	6.4201		33.8182	5.8849	2.8273	63.003			1.0479	0.0115	81.5373	11.4756	0.029
91	1-methyl-4-(5-methyl-1-methylene-4-hexenyl)-Cyclohexene																0.0462									
92	1-Methyl-5-(1-methylethenyl)-Cyclohexene,																0.1947									
93	3-methyl-6-(1-methylethylidene)cyclohexene			0.1718																						
94	4-Methylene-1-(1-methylethyl)-cyclohexene	1.9176		2.135		2.2519		1.8514									0.0739		1.4432				1.9632	1.9308		
95	3-isopropenyl-5,5-dimethyl-Cyclopentene															3.8759										
96	4-ethenyl-1,5,5-trimethyl-Cyclopentene	0.2091																								
97	1-(2-Propenyl)-cyclopentene																									0.1364
98	octamethyl-Cyclotetrasiloxane															1.0671										
99	Hexamethyl-Cyclotrisiloxane							0.9918																	0.8073	
100	D-Limonene																			0.6041						0.3268
101	Eicosane																	0.2245								
102	1,2-Bis(2-chloroethoxy)-ethane					0.1557																				0.0797
103	4'-Methylacetophenone					0.0438																				
104	Heneicosane																	0.4635								
105	Hexanoic acid,3-hexenyl ester			0.0445												0.004										
106	Limonene				0.2566													1.5074			0.9416					
107	Longifolene-(V4)															0.0789										0.679
108	1,2,3,4,4a,5,6,8a-octahydro-4a,8-dimethyl-2-(1-methylethenyl)-, [2R-(2.alpha.,4a.alpha.,8a.beta.)]-Naphthalene								0.2373							0.183	0.1038		5.3282			17.7823		0.3419		
109	1,2,3,4,6,8alpha-Hexahydro-1-isopropyl-4,7-dimethylnaphthalene																									0.0674
110	Valencene																		0.2722					0.232		
111	1,2,3,5,6,7,8,8a-octahydro-1,8a-dimethyl-7-(1-methylethenyl)-, [1S-(1.alpha.,7.alpha.,8a.alpha.)]-Naphthalene															0.0144										
112	1,2,3,5,6,8a-hexahydro-4,7-dimethyl-1-(1-methylethyl)-(1S-cis)-Naphthalene					0.0487	0.0252		0.1824				0.7626				0.1352					1.4689				1.5862
113	1,2,4a,5,6,8a-hexahydro-4,7-dimethyl-1-(1-methylethyl)-Naphthalene					0.0692																				1.8182
114	α-Muurolene					0.1311	0.0471																			
115	1,2,4a,5,8,8a-hexahydro-4,7-dimethyl-1-(1-methylethyl)-, [1S-(1.alpha.,4a.beta.,8a.alpha.)]-Naphthalene																									
116	β-Cadinene	0.3401				0.4812	0.1401																0.0059			
117	decahydro-4a-methyl-1-methylene-7-(1-methylethenyl)-, [4aR-(4a.alpha.,7.alpha.,8a.beta.)]-Naphthalene				3.0895				1.9284				2.7228			1.0316		1.2042			3.3372			3.9186		7.895
118	Decahydro-4a-methyl-1-methylene-7-(1-methylethenyl)-, [4aR-(4a.alpha.,7.alpha.,8a.beta.)]-Naphthalene							7.499						0.3306												
119	γ-Selinene																									2.6824
120	Palmitic acid						0.0282																			
121	1-Nonanal			0.0467																						
122	2,6-Bis(1,1-dimethylethyl)-4-(1-methylpropyl)-phenol																						0.0088			
123	4,4'-(1-methylethylidene)bis-Phenol			0.1716						12.1471			1.4004					2.7819			0.5711					0.1058
124	Phthalic acid, isobutyl octyl ester					1.28								2.9376												
125	2-methyl-Propanoic acid, 3,7-dimethyl-2,6-octadienyl ester					0.0497																				
126	2-methyl-Propanoic acid,hexyl ester			0.0267									0.201	0.0314		0.2107								0.036		
127	p-Trimethylsilyloxyphenyl-bis(trimethylsilyloxy)ethane										0.849															

Note: Values correspond to relative volumetric amounts of individual components.

### Different volatile compositions and proportions in the 25 mango pulps

The significant differences in the compositions and proportions of the volatile components in the 25 mango pulps are shown in Table 3. Monoterpenes, some of the primary volatile components, were the most abundant aroma constituent, whereas aldehydes were the least common volatile constituents in the mango pulps, ‘Tommy Atkins’ had the highest monoterpenes content of 97.48%, followed by ‘Renong No.1’, ‘Gleck’ and ‘Baodaohuang’, which all had content of more than 91%. The aldehydes content were lowest in these mango pulps (Table 3).

**Table 3:** Relative contents of various chemical classes in different mango cultivars.

Cultivar code	Volatile compounds (%)										
	M	S	Nt	L	Alc	Ald	K	Ac	Alk	P	O
1	87.92	4.46	1.12	1.15	0.25		0.20		0.21		0.98
2	2.41										14.84
3	92.26	3.57	0.12	0.71		0.38					14.84
4	85.43	6.59		1.27			1.16				
5	89.32	2.93		2.31			0.16		0.16		0.13
6	49.23	1.34	0.17	0.78			0.01	0.91		0.08	
7	81.11	10.32							0.99		
8	97.48	12.02									
9	18.60		1.68	6.84						12.15	15.39
10	17.82		71.18						0.85		4.98
11	27.48										
12	69.53	12.74	1.21	2.11						1.4	0.37
13	7.03	0.33		4.75	0.12						
14		22.20									4.63
15	61.33	1.60	13.72	2.53			0.05		1.07		4.38
16	73.66	20.44	0.19								1.97
17	73.87	2.46	0.221		0.42		3.28		0.69	2.78	
18	72.65	6.09									
19	0.69										
20	83.38	8.02		0.57						0.57	
21	1.05	22.41									
22	91.21	1.41	0.02	0.15		0.01					0.04
23	91.50	5.20	0.41	1.35							0.35
24	11.48			25.85					.081		32.4
25	81.53	15.82	1.00	0.34			0.05		0.08	0.11	1.97

Note: M, S, Nt, L, Alc, Ald, K, Ac, O, Alk and P indicate monoterpene, sesquiterpen, non-terpene, lactones, alcohols, aldehydes, ketones, acids, others, alkanes, phenols, respectively. The same below. Values corresponds to the relative volumetric amounts, where volatile compounds = Ʃ(score of each component belonging to the same compound).

### Correlation analysis of the volatiles

The highly significant positive correlations between volatiles alcohols (Alc) and ketones (K), alkanes (Alk) and non-terpene (Nt), O (others) and lactones (L), detected with a correlation analysis, are noteworthy. However, other pairs of volatile components showed negative correlations, such as Nt and monoterpene (M) or sesquiterpene (S), L and M, S, or Nt, etc., although these were not significant (Table 4).

**Table 4:** Correlation analysis of the volatiles.

Indexes	M	S	Nt	L	Alc	K	Alk	P	O
M	1	0.090	-0.199	-0.281	0.125	0.188	0.086	-0.161	-0.362
S	——	1	-0.206	-0.274	-0.168	-0.112	-0.189	-0.181	-0.279
Nt	——	——	1	-0.074	-0.075	-0.069	0.521**	-0.046	0.035
L	——	——	——	1	-.061	-0.090	-0.075	0.167	0.797**
Alc	——	——	——	——	1	0.795**	0.264	0.109	-0.157
K	——	——	——	——	——	1	0.282	0.143	-0.147
Alk	——	——	——	——	——	——	1	-0.051	-0.078
P	——	——	——	——	——	——	——	1	0.266
O	——	——	——	——	——	——	——	——	1

Note:–means negative correlation;

** significant correlation (*P<*0.01);——none.

### Cluster analysis of volatile components

**Fig 2:**
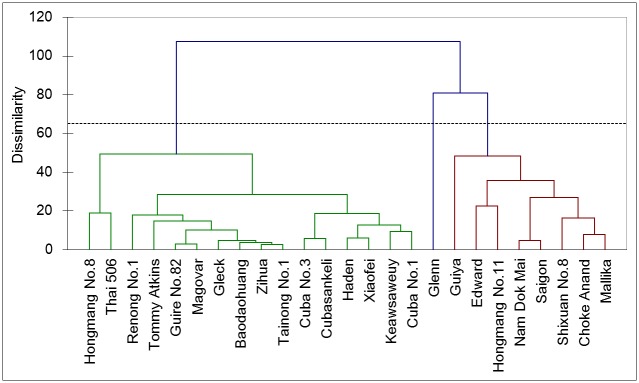
Cluster dendrogram of 25 mango cultivars.

A cluster analysis was conducted based on the volatile composition and proportion data for the 25 cultivars, and the cluster dendrogram was presented in Fig 2. The results show that, at 50% dissimilarity, cultivar 6, 15, 3, 8, 4, 20, 22, 23, 1, 5, 17, 18, 7, 25, 12, and 16 were analogous in their volatile compositions and proportions, with diverse volatile compositions and containing mainly monoterpenes (these cultivars were grouped as the ‘monoterpenes’ group). Cultivars 24, 9, 11, 14, 21, 2, 13, and 19 clustered together, with lower volatile compositions and proportions (these cultivars were grouped in the ‘proportion and balanced’ group). Interestingly, cultivar 10 was classified alone based on its unique lack of terpene (this cultivar was independently classified in the ‘nonterpene’ group).

### Regional differences in mango volatiles

**Fig 3:**
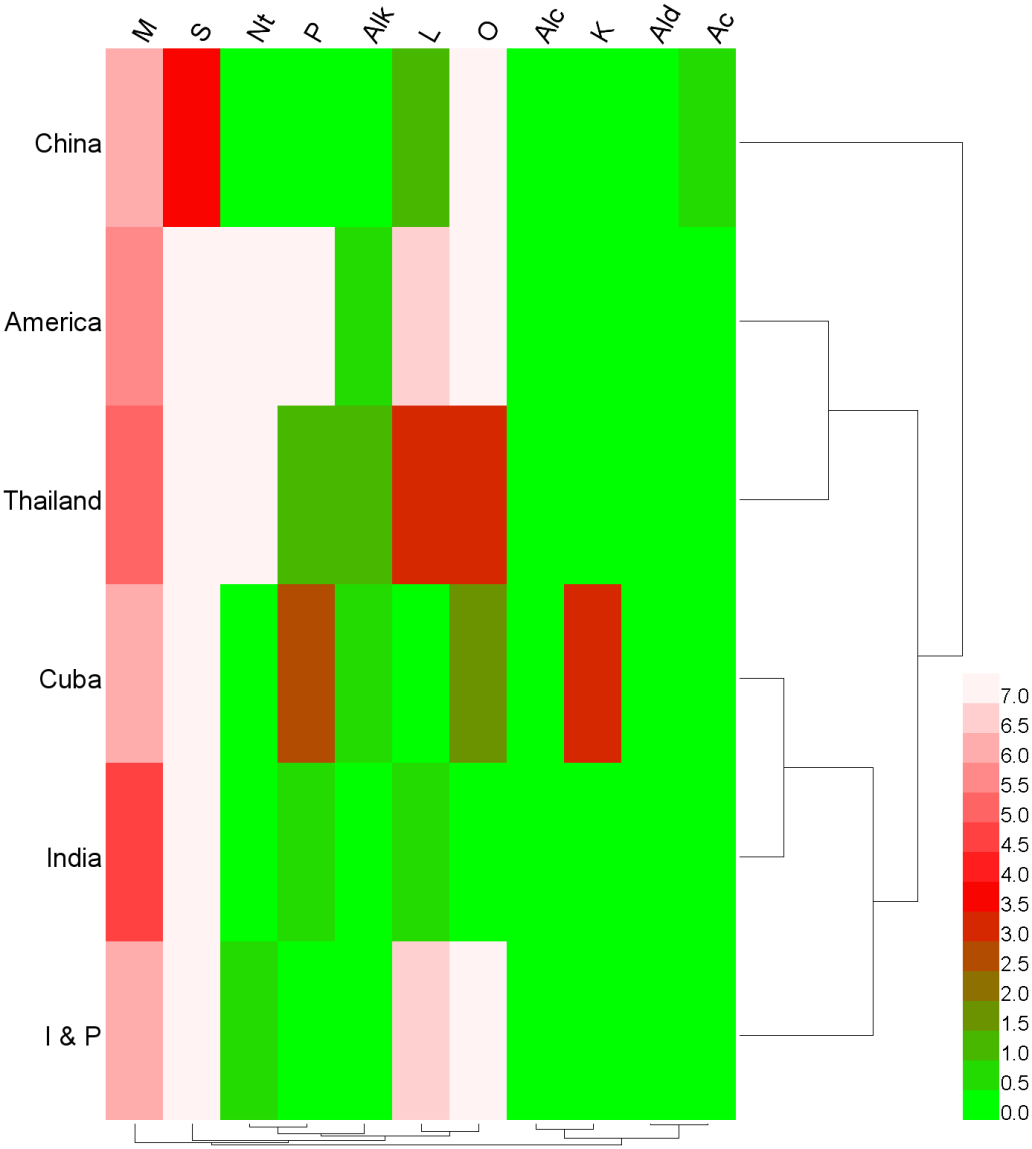
Cluster dendrogram of six regions. “I & P”, Indonesia and the Philippines; M, monoterpene; S, sesquiterpene; Nt, nonterpene; P, phenols; Alk, alkanes; L, lactones; Alc, alcohols; K, ketones; Ald, aldehydes; Ac, acids and O, others.

To clarigy the variations in the cultivars originating from seven distinct regions, a cluster analysis was performed base on their volatile contents. The results suggest that the cultivars originating from America, Thailand, Cuba, India, Indonesia and Philippines were more similar to one another than to those from China. The cultivars originating from America and Thailand, as well as Cuba and India, were especially similarity (Fig 3).

Monoterpenes were again the most common volatiles, which is consistent with the results in Table 3. Individually, the cultivars originating from India contained the most monoterpene, followed by those originating from America and Thailand, whereas the cultivars originating from China, Cuba, Indonesia, and the Philippine contained the least monoterpenes (Fig 3). The cultivars originating from China had the highest contents of sesquiterpen, Whereas those originating from Cuba had the highest contents of both phenols and ketones, and those from Thailand had the most monoterpenes, lactones, and other volatiles.

## Conclusion

The volatile components that confer the aroma of fruit are one of the most important indicators of quality. Our analysis of the volatile organic compounds in mango pulp allowed us to differentiate mango cultivars and indicated the complex synthetic mechanisms involved in the production of their aroma [22]. Altogether, 127 volatiles were detected in all the selected mango cultivars, which belonged to various chemical classes. The highest and lowest qualitative abundance of volatiles were observed in the ‘Zihua’ and ‘Mallika’ cultivars, respectively.

Previous research by Pino et al. indicated that terpenes are the primary aroma constituents of mangoes [10]. Andrade et al. divided mango cultivars into terpinolene cultivars, 3-carene cultivars, and myrcene cultivars, based on their different constituent terpenes [19]. Similar to previous studies, in the present study, we found that terpenes were the main aroma constituents of 16 mango cultivars, and included α-pinene, selinene, caryophyllene, careen, and terpinolene. However, we detected significant differences in terpenes compositions and proportions in the 25 cultivars: the terpenes contents of ‘Guire No. 82’, ‘Magovar’, ‘Gleck’, ‘Baodaohuang’, ‘Cuba No. 3’, and ‘Cubasankeli’ were much higher than those of ‘Shixuan No. 8’, ‘Choke Anand’, ‘Mallika’, and ‘Edward’ (Table 3). ‘Shixuan No. 8’ had only two major aroma components, terpinolene and squalene, and was the only cultivar to contain squalene, with a content of 43.91%. Liu et al. [23] reported that selinene, eremophilene, and aromadendrene were only detected in ‘JinHwang’; limonene were detected in ‘Irwin’; and α-caryophyllene were detected in ‘Keitt’. Interestingly, in this study, ‘Thai 506’ was the only cultivar to contain squalene, though the content was only 0.01%. Although a few β-squalene-, limonene-e, or α-caryophyllene-containing cultivars were found in this study, no cultivar contained aromadendrene. These large differences may be attributable to differences in the cultivation conditions, maturity levels, and postharvest storage treatments, as well as to the cultivars themselves. In these ways, differences in aroma and flavor are generated in mango fruit.

Aldehyde occurs at a low level, but plays a key role in mango flavor. This observation was supported by the earlier finding by Macleod et al. that the aldehydes content of mangoes was 0.03%-14.36% [24]. In the present study, only two cultivars contained aldehydes. However, Pino et al. [10] considered that the aldehydes in Cuban mango cultivars was associated with the sweet herbal flavor of their fruit. A typical aldehydic aroma, 2,6-di-tert-butyl-4-sec-butylphenol, was detected in ‘Gleck’ in the present study.

Aldehydes are easily broken down to alcohols and lactones [25]. Alcohols occur in small amounts in mango fruit and contribute slightly to their aroma and flavor [26,27]. In this study, only three cultivars contained alcohols. Lactones confer a full-bodied fruit aroma, and Andrade et al.[19] reported lactones to be the second most common aroma volatile in the mango. A study by Wilson et al. [28] suggested that lactones occur at low but still detectable level in mango fruit, and Pandit et al. [29] found eight lactones in 18 mango cultivars. γ-Octanoic lactone was the only ester detected among the total flavor volatile compounds in the present study, with its highest concentration in ‘Guiya’ (China).

The cluster analysis of regional difference was limited in this study by the partial and small number of specimens in the mini-core collection of mango germplasms. Therefore, further analysis should be conducted with more mango germplasms in the future. The highly significant positive correlation and other negative pairwise correlations among the volatile components suggest that metabolic interrelationships and the regulation of the volatiles in mango pulp warrant further research to assist the development and utilization of mango aromas.
